# Effect of Aqueous Cinnamon Extract on the Postprandial Glycemia Levels in Patients with Type 2 Diabetes Mellitus: A Randomized Controlled Trial

**DOI:** 10.3390/nu14081576

**Published:** 2022-04-10

**Authors:** Ana Paula Rachid, Margarida Moncada, Maria Fernanda de Mesquita, José Brito, Maria Alexandra Bernardo, Maria Leonor Silva

**Affiliations:** Centro de Investigação Interdisciplinar Egas Moniz, Instituto Universitário Egas Moniz, Campus Universitário, Quinta da Granja, 2829-511 Monte de Caparica, Portugal; rachid.anapaula@gmail.com (A.P.R.); margaridacm@egasmoniz.edu.pt (M.M.); fmesquita@egasmoniz.edu.pt (M.F.d.M.); britojaa@hotmail.com (J.B.); abernardo@egasmoniz.edu.pt (M.A.B.)

**Keywords:** *Cinnamomum burmannii*, cinnamon, postprandial glycaemia, type 2 diabetes mellitus, antioxidant, polyphenols

## Abstract

Cinnamon is a spice used in traditional cuisine that has been investigated due to hypoglycemic properties. The objective of this study was to investigate the effect of aqueous cinnamon extract on postprandial glycemia levels in type 2 diabetes mellitus (DM2) adults. This clinical trial enrolled 36 adults with DM2, randomly allocated in two groups: the control group (*n* = 18) took only an oral glucose tolerance test (OGTT) and the intervention group (*n* = 18) took OGTT immediately followed by aqueous cinnamon extract (6 g/100 mL) ingestion. Blood glucose levels were measured on fasting and after 30, 60, 90 and 120 min in both groups. The chemical analysis of the aqueous cinnamon extract included total phenols content determination and antioxidant activity assessment through FRAP and DPPH methods. The data reveal that aqueous cinnamon extract ingestion did not show a significant difference in the incremental area under the curve (*p* = 0.834), maximum glucose concentration (*p* = 0.527) and glucose concentration variation (*p* = 0.873) compared with the control group. Cinnamon extract possess a total phenol content of 1554.9 mg/L gallic acid equivalent and a strong antioxidant capacity, revealed by the DPPH (5125.0 µmol Trolox/L) and FRAP (3658.8 µmol Trolox/L) tests. Aqueous cinnamon extract did not significantly influence postprandial glucose response in diabetic patients during an OGTT.

## 1. Introduction

Type 2 diabetes mellitus (DM2) is characterized by high levels of blood glucose due to relative insulin deficiency caused through pancreatic beta-cell dysfunction and insulin resistance. Globally, the incidence and prevalence of DM2 continues to rise, with the number of patients with DM2 estimated to increase worldwide to 592 million by 2035 [1,2,3]. Epidemiological studies have showed that postprandial glucose levels control is important since it is associated with cardiovascular complications in type 2 diabetic mellitus patients [2].

The use of traditional plants as complementary therapies has been growing due to their effect on health, including antioxidant and anti-inflammatory activity as well as their effect against diabetes mellitus and cardiovascular disease [4,5,6,7]. Cinnamon is an indigenous spice from the genus Cinnamomum that has shown several beneficial functional properties in health, including a protective activity in DM2 [8,9,10,11]. Cinnamon burmannii tea was revealed to significantly lower postprandial maximum glucose concentration and variation in maximum concentration in healthy adults [12]. Furthermore, cinnamon burmannii powder added to a high-sugar dessert significantly reduced postprandial glucose levels (PBG) area under the curve [13,14], and Cinnamomum cassia powder capsule (2 g per day) intake over a 40-day period significantly reduced PBG by 12.8% (*p* < 0.01) [15]. Another study reported that the ingestion of cinnamon bark powder (500 mg capsules twice daily) for 3 months significantly lowered (*p* < 0.001) 2-hour PBG compared with the control group [16]. Studies have also demonstrated that cinnamon exerts a beneficial effect in fasting blood glucose (FBG) among diabetic patients [16,17,18,19]. Sahib et al. suggest that cinnamon intake (1 g) for 12 weeks can significantly (*p* < 0.05) contribute to FBG reduction and also to oxidative stress improvement among poorly controlled DM2 patients [20]. However, the current evidence suggests that more controlled trials about postprandial glycemic parameters in diabetic patients should be conducted due to the poor quality of studies [21].

The objective of this study was to investigate (1) the effect of aqueous cinnamon extract (6 g cinnamon burmannii/100 mL) on postprandial glycemia levels in type 2 diabetic adults and (2) the total phenols content and antioxidant evaluation.

## 2. Materials and Methods

### 2.1. Participants

Type 2 diabetes mellitus (DM2) subjects aged between 35 and 77 years were recruited into this study between January 2017 and October 2017 at a nutrition appointment from Holon Pharmacy from Lisbon and Portalegre, Portugal. A total of 36 individuals were selected and invited to participate in this study. The inclusion criteria included subjects aged 18 years or older, subjects with DM2 diagnostic and men or non-pregnant women. Exclusion criteria included insulin-treated subjects, history of gastrointestinal symptoms/diseases, allergy to cinnamon, fasting less than 8 h, cinnamon intake on the day before the intervention and intense exercise 2 h before the intervention. This study was approved by the Ethical Committee of Cooperativa de Ensino Superior Egas Moniz (425 process; approval 22 October 2015) and was carried out according to the Declaration of Helsinki. The anonymity and the confidentiality of the participants collected data were guaranteed through a code assigned to each participant. After oral and written information about the aim and procedures of the study, an informed consent was given and signed by all participants. This clinical trial is registered at Clinicaltrials.gov (NCT05140629).

### 2.2. Study Design

After eligibility criteria were applied, a randomized controlled clinical trial, blind to participants, was conducted with 36 DM2 subjects. Participants were randomly assigned to intervention (*n* = 18) or control group (*n* = 18). The first participant of the study was randomly allocated to the intervention or control group and the subsequent participants were systematically allocated in each group.

The control group was given only a glucose solution for an oral glucose tolerance test (OGTT) and the intervention group was given a glucose solution for an OGTT immediately followed by a cinnamon aqueous extract. The glucose solution consisted in a glucose drink with 75 g of anhydrous D-glucose dissolved in 200 mL of water at room temperature, as prescribed by the ADA [22]. The blood glucose levels were measured before the intervention at fasting (t0) and after 30 (t30), 60 (t60, 90 (t90) and 120 (t120) minutes after intervention in both groups.

**Figure 1:**
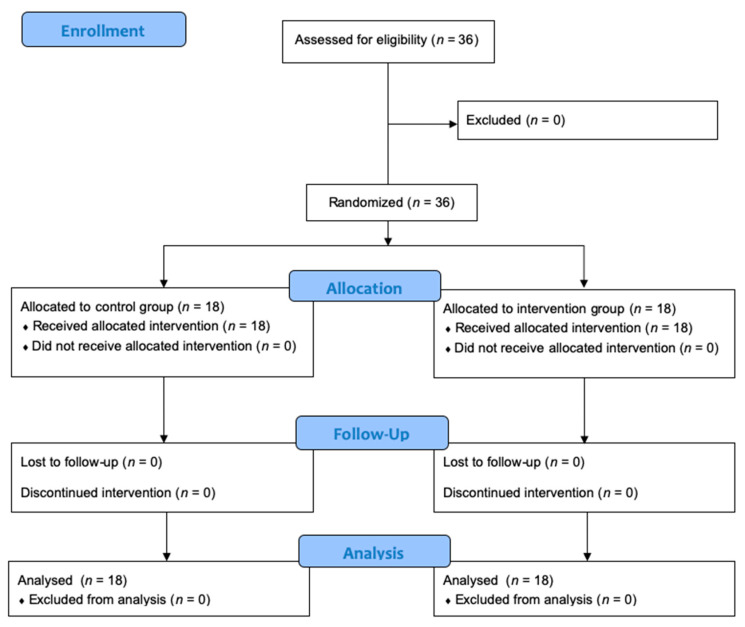
CONSORT flow diagram of the study.

The flow diagram enrolment, allocation, follow-up and analysis of this study participants are showed in Figure 1.

### 2.3. Aqueous Cinnamon Extract Preparation

The *Cinnamomum burmannii* bark was purchased from the Sucrame Company (Portugal) with Indonesia as its origin. Sticks of cinnamon (60 g) were soaked in 1000 mL of water. After 24 h at room temperature, the cinnamon solution was heated for 30 min at 100 °C and then filtered at room temperature. This method was adapted by Shen and coauthors [23]. After aqueous cinnamon extract preparation, a 100 mL individual dose was distributed to each participant in the intervention group. The aqueous cinnamon extract concentration was obtained through determination of lyophilized extract weight.

For chemical analysis, a hydromethanol extract (50:50) was performed with aqueous cinnamon extract previously obtained [12].

### 2.4. Body Composition and Clinical Assessment

Demographic information, anthropometric paraments and pharmacological therapy were collected by trained interviewers at the beginning of the study. Regarding anthropometric parameters, the body weight (kg), body fat mass (%), skeletal muscle mass (kg) and visceral fat (cm^3^) were estimated by bio-impedance through a Tanita^®^ scale (model BC-545N). The body mass index (BMI) was calculated as the ratio of body weight (kg) and height (m^2^) squared (kg/m^2^).

### 2.5. Dietary Intake Assessment

At day before the intervention, a 24-hour food recall questionnaire was administered to participants of the study in order to verify the homogeneity on dietary parameters between groups. The questionnaire was carefully instructed by an investigator to complete the food record. Additionally, food amounts were estimated using a picture book in order to estimate the portion sizes of meals [24].

The Food Processor SQL (10.14.2. version) program was applied to analyze the nutritional composition of meals, such as, total energy (Kcal), total proteins (g), total fat (g), total carbohydrates (g), dietary fiber (g) and soluble fiber (g). The glycemic index and glycemic load of food intake were also estimated.

### 2.6. Blood Glucose Level Analysis

Capillary blood samples were collected from each participant to measure plasma glucose levels. Sterile lancets, glucose meter equipment (On Call Extra) and test strips (FreeStyle_Abbott Diabetes Care) for the glucose meter were used in order to measure blood glucose. Based on the blood glucose values, the blood glucose incremental area under the curve (AUCi) of each participant was estimated using the GraphPad Prism program (version 5.0). Maximum concentration (Cmax) and variation of maximum concentration (ΔCmax) were determined by comparing respective baseline glycemia level values.

### 2.7. Characterization of Antioxidant Capacity

#### 2.7.1. Chemical Analysis Reagents

Ferric chloride (III) hexahydrate (FeCl_3_·6H_2_O), Folin–Ciocalteu, Trolox (6-hydroxy-2,5,7,8-tetramethylchroman-2-carboxylic acid), TPTZ 2,4,6-tri(2-pyridyl)-s-triazine, DPPH (2,2-diphenyl-1-picrylhydrazyl) and methanol (CH_3_OH) were purchased from Sigma-Aldrich; gallic acid-1-hydrate (C6H2(OH)3COOH·H_2_O) was purchased from Acros Organics, and sodium carbonate (Na_2_CO_3_) was purchased from ICS Science group. All reagents were pro analysis grade. The reagents were weighed in an analytical balance (Sartorius ± 0.0001 g).

#### 2.7.2. Total Phenolic Content Determination

The total phenolic concentration in the C. burmannii extract was determined according to Folin–Ciocalteu method [25], employing gallic acid as standard. The results are expressed as mg for gallic acid equivalent (GAE)/g of extract. A volume of 375 μL of cinnamon extract and 4 mL of sodium carbonate was added to 5 mL of Folin–Ciocalteu reagent. After 15 min, the absorbance was measured at 765 nm.

#### 2.7.3. Antioxidant Activity Assay

For the antioxidant activity analysis, two methods were performed: FRAP (ferric reducing antioxidant power) and DPPH (2,2-diphenyl-1-picrylhydrazyl) adapted by Thaipong et al. [26].

The FRAP method was determinated through a ferric reducing effect based on blue-colored ferrous complex (Fe^2+^) formation by electron-donating antioxidants action in 2,4,6-tri(2-pyridyl)-s-triazine (TPTZ) presence. A fresh solution was prepared by mixing 25 mL of acetate buffer (300 mM, pH = 3.6) into 2.5 mL of TPTZ solution (10 mM) in HCl (40 mM) and 2.5 mL of FeCl_3_·6H_2_O solution (20 mM). The solution was heated at 37 °C. Samples (150 μL) were introduced in tubes with 2850 μL of the FRAP solution and were maintained under dark conditions for 30 min. The absorbance was measured at 593 nm. Trolox (6-hydroxy-2,5,7,8-tetramethylchroman-2-carboxylic acid) was used as standard, and the results are expressed in μmol Trolox/L.

The DPPH method was determined through 2,2-diphenyl-1-picrylhydrazyl radical scavenger [26]. A volume of 10 mL of solution prepared (24 mg DPPH in 100 mL methanol) was added to 45 mL of methanol (λ = 515 nm Abs 1.1). The solutions were kept for 24 h in the absence of light. The absorbance was determined at 515 nm, and the results are expressed in µmol Trolox/L.

### 2.8. Statistical Analysis

Statistical analysis was performed using SPSS^®^ software (Statistical Package for Social Sciences) version 27.0 software for Mac. Descriptive analysis data are reported as the mean ± SEM. The difference between groups in postprandial blood glucose at different times was assessed using repeated measures ANOVA of mixed type. After assumption verification, differences between the groups in anthropometric parameters and dietary parameters were assessed using independent samples t-test or Mann–Whitney test. The difference between groups for Cmax, ΔCmax and AUCi was assessed using the Mann–Whitney test. All statistical tests were performed at the 5% level of significance.

## 3. Results

### 3.1. Baseline Sample Characterization

A total of 36 participants with type 2 diabetes mellitus completed the study: 18 subjects in the intervention group (3 male, 15 female) and 18 subjects in the control group (7 male, 11 female). There were no significant differences (*p* > 0.05) between control and intervention group for age and anthropometric parameters, Table 1. Regarding pharmacological therapy, more than 50% of the participants in both groups were medicated with oral antidiabetics—namely, biguanides (Table 1). Globally, the participants of the study presented overweight or obesity (94.4%), according to BMI classification [27].

At the day before the intervention, no significant differences (*p* > 0.05) were observed between the control and intervention group regarding dietary patterns, glycemic index and glycemic load (Table 2), which means that the two groups were considered homogeneous.

### 3.2. Postprandial Blood Glucose Levels

The mean blood glucose levels for the intervention and control groups are shown in Table 3. The analysis of independent factors of repeated measures (ANOVA) revealed that there was no interaction between the independent and repeated measures factors (*p* = 0.870), which means that differences in postprandial blood glucose in different moments are the same in both groups and that differences between groups do not change with time.

The blood glucose AUCi over 120 min period revealed that the ingestion of aqueous cinnamon extract following glucose load had a slight lowering effect on glucose response, which was not significant between groups (*p* = 0.834). There was also no significant difference in Cmax (*p* = 0.527) and ∆Cmax (*p* = 0.873) between groups, Table 4.

### 3.3. Antioxidant Activity of Aqueous Cinnamon Extract

Chemical analysis revealed that aqueous cinnamon extract possesses a total polyphenols content of 1554.9 mg/L. Moreover, the extract of *C. burmannii* showed a strong antioxidant activity according with FRAP assay and DPPH assay, Table 5.

## 4. Discussion

The results of this study show that the ingestion of aqueous cinnamon extract (6 g) has no significant effect on postprandial glycemia over time in patients with DM2 (*p* = 0.870) compared with the control group.

This result is in agreement with other authors demonstrating that there are no significant differences in postprandial glycemia between groups in adult subjects with DM2 with lower doses of cinnamon of 1, 1.5 or 3 g daily [6,28,29,30]. However, other works, where the effect of cinnamon on postprandial glycemia in diabetic subjects is studied, revealed controversial results. Cinnamon capsules (1 to 6 g) seemed to exert a beneficial effect in postprandial glycemia in DM2 patients after 1 to 3 months intervention compared with baseline [18,19,31]. The effect of cinnamon is possibly effective in diabetic subjects after a longer time of supplementation, suggesting that a short-time is not significant in altering glycemic parameters.

The different plant materials including bark powder and water extracts as well as doses and formulation could also contribute to the heterogeneity in clinical trial results. The Hayward et al. study showed that antidiabetic properties of cinnamon also depend on species. According to this study, Ceylon cinnamon was demonstrated to be more effective on anti-hyperglycemic properties [32].

In addition to these multiple factors, differences in current medication may also influence the cinnamon action in glucose metabolism. The study design considered the initial drug monotherapy (biguanides) or the two-drug combinations (biguanides plus other oral antidiabetic), excluding patients with three-drug combinations or more complex insulin strategies. However, the diversity of sample drugs can contribute as a potential bias. In this context, this confounding factor should be adjusted in further studies. Furthermore, the authors suggest that the age range should be reduced in order to obtain more reliable results.

The authors point out that the diet style of the subjects was not evaluated in the present study, which could be pertinent in evaluations in further studies in order to better understand diet’s influence in the outcome measured.

Zare et al. (2019) showed that DM2 patients with BMI < 27 and BMI > 27 taking cinnamon capsules (500 mg) twice daily for 3 months significantly improved two-hour postprandial blood glucose levels, reporting that BMI may not be a predictor of response in diabetic patients [16].

Our results also reveal that cinnamon had no significant effect in glucose response AUCi, maximum glucose concentration (Cmax) and variation in glucose levels (ΔCmax) between groups, which are in accordance with a Wickenberg et al. study [33] reporting that 6 g of *C. zeylanicum* cinnamon capsules followed by an OGTT did not influence glucose response.

We suggest that cinnamon is probably effective in diabetic patients as a part of usual mixed-meals. According to a Hayward et al. study, cinnamon powder showed an inhibition of starch digestion enzyme activity, which resulted in significantly decreased starch breakdown during digestion [32]. Furthermore, cinnamon extract also seemed to act by regulating glucose metabolism thought hepatic gluconeogenesis regulation. Specifically, cinnamon ingestion decreased the gene expression of phosphoenolpyruvate carboxykinase (PEPCK) and glucose-6-phosphatase [34]. Additionally, cinnamon exerted a beneficial effect on glycemia by hepatic glycogen synthesis through glycogen synthase kinase inhibition [35]. In addition, the insulin-mimetic effect seems to also contribute to the hypoglycemic properties of cinnamon extract. In a Shen et al. study, cinnamon extract was shown to increase glucose uptake in muscle and adipose tissues through glucose transporter (GLUT) 4 production and GLUT 4 translocation [23,36].

Considering the heterogeneity and conflicting nature of study results regarding the effect of cinnamon on glycemia, cinnamon supplementation should not be recommended generically in DM2 management. Furthermore, taking into account that diabetes mellitus is also usually diagnosed based on HbA1C, this parameter should be measured in order to better understand the effect of cinnamon in this disease. Insulin-level analysis should also contribute to verification of cinnamon’s impact on diabetes, suggesting its inclusion in further studies.

In the present study, we also determined the total phenol content since the bioactive compounds in cinnamon have been associated with its therapeutic properties [37]. The major chemical compounds responsible for the beneficial effect of cinnamon in health are polyphenols, including, procyanidin type-A polymers, cinnamic acid, cinnamaldehyde and coumarin [11,38,39,40,41]. The polyphenolic content in the present aqueous cinnamon extract (1554.9 ± 72.8 mg/L gallic acid) was approximately half compared with other aqueous extracts of cinnamon (2286.3 ± 48 mg/L gallic acid [12]). Taking into account that cinnamon polyphenols seem to exert hypoglycemic activity by increasing GLUT 4 accumulation in 3T3-L1 adipocytes, this could be one of the explanations for the nonsignificant effect on glucose response in this study [42]. According to the literature, the polyphenolic content obtained in our cinnamon extract could be due to cinnamon composition, which depends on different factors, such as tree section (top, center and lower segments) and on different growth stages. According to a Geng et al. study, the bark products have a minimum of cinnamaldehyde at one years’ growth (33.95%) and a maximum at six years’ growth (76.4%) [43]. Another possible reason could be related to the method of cinnamon extraction.

The antioxidant activity obtained in our aqueous cinnamon extract by DPPH assay (5125.0 ± 74.3 µmol Trolox/L) was also lower compared with a Bernardo et al. study (11,853.4 ± 322.8 µmol Trolox/L) [12]. This result is related to the polyphenol compounds content obtained in the extract. According to a Dudonné et al. study, antioxidant activity is significantly correlated with its polyphenolic content [44], demonstrating a relationship between these bioactive compounds and their free radical scavenging and ferric-reducing capacities.

A recent meta-analysis showed that 1.5 to 4 g of cinnamon powder significantly increased total antioxidant capacity and decreased interleukin, an inflammatory biomarker, suggesting that cinnamon may be a coadjutant for oxidation and inflammation management in humans [45]. In patients with impaired fasting glucose, daily ingestion of aqueous cinnamon extract capsules (500 mg) for 12 weeks significantly improved plasma oxidative stress markers [46].

Taking in account that oxygen reactive species (ROS) and oxidative stress have been reported as the main factors for impaired insulin secretion, glucose mobilization and consequently DM2 [47], cinnamon could be used as a coadjutant for oxidative management preventing diabetes. According to a previous study, *C. burmannii* has the highest polyphenol content and significantly higher antioxidant properties compared with other species of cinnamon [32].

## 5. Conclusions

Aqueous cinnamon burmannii extract (6 g/100 mL) ingestion did not significantly change the postprandial glycemia values in individuals with DM2 compared with the control group; in spite of this, cinnamon extract possesses a considerable antioxidant activity and inhibition capacity of reactive oxygen species. Therefore, the present study encourages the intake of aqueous cinnamon extract as a source of natural antioxidants due to its high content in these compounds and respective antioxidant activity.

Further studies with a larger sample size should be employed over a longer period to test the effects of cinnamon burmannii extract as part of mixed-meal daily intake.

## Figures and Tables

**Table 1:** Baseline characteristics of the participants for intervention (*n* = 18) and control (*n* = 18) groups.

Parameters	Intervention Group Mean ± SEM	Control Group Mean ± SEM	*p*-Value
Age, years	63.5 ± 1.6	62.06 ± 2.4	0.625 ^1^
Body weight, kg	84.3 ± 3.1	79.8 ± 3.6	0.319 ^2^
Height, m	1.6 ± 0.02	1.6 ± 0.02	0.824 ^2^
Waist circumference, cm	110.2 ± 1.7	105.3 ± 2.5	0.051 ^2^
Body mass index, kg/m^2^	32.5 ± 1.0	31.0 ± 1.3	0.223 ^2^
Body fat mass, %	38.8 ± 1.3	37.7 ± 1.9	0.354 ^1^
Skeletal muscle mass, kg	48.3 ± 1.9	46.9 ± 2.0	0.725 ^1^
Visceral fat, cm^3^	15.0 ± 1.4	13.2 ± 0.9	0.657 ^2^
Pharmacological therapy	*n* (%)	*n* (%)	
Biguanide	14 (77.8)	16 (88.9)	
Sulfonylurea	7 (38.9)	7 (38.9)	
Alpha-glucosidase inhibitor	0	1 (5.6)	
Dipeptidyl peptidase 4 (DPP4) inhibitors	2 (11.1)	0	
Biguanide + Dipeptidyl peptidase 4 (DPP4) inhibitors	2 (11.1)	7 (38.9)	

^1^*p*-value calculated by independent samples t-test between groups. ^2^*p*-value calculated by Mann–Whitney test between groups.

**Table 2:** Mean (± SEM) values of the dietary parameters on the day before the intervention for intervention (*n* = 18) and control (*n* = 18) group.

Dietary Parameters	Intervention Group Mean ± SEM	Control Group Mean ± SEM	*p*-Value
Total energy, Kcal	2090.24 ± 223.28	2340.46 ± 249.09	0.467 ^2^
Total protein, g	88.56 ± 12.52	104.53 ± 11.59	0.174 ^2^
Total fat, g	52.67 ± 6.50	50.39 ± 7.64	0.613 ^2^
Total carbohydrate, g	302.43 ± 30.99	356.06 ± 41.40	0.383 ^1^
Dietary fiber, g	34.44 ± 4.72	44.78 ± 6.14	0.192 ^1^
Soluble fiber, g	2.55 (± 0.71)	1.14 ± 0.27	0.383 ^2^
Glycemic index	48.10 ± 4.60	43.68 ± 2.23	0.939 ^2^
Glycemic load	19.00 ± 6.07	10.09 ± 2.46	0.544 ^2^

^1^*p*-value calculated by independent samples t-test between groups. ^2^*p*-value calculated by Mann–Whitney test between groups.

**Table 3:** Mean blood glucose levels (mmol/L) obtained for control (*n* = 18) and intervention group (*n* = 18) at different moments: before intervention (t0) and 30 (t30), 60 (t60), 90 (t90) and 120 (t120) minutes after intervention.

Time	Intervention Group Mean ± SEM (mmol/L)	Control Group Mean ± SEM (mmol/L)
t0	8.10 ± 0.75	7.02 ± 0.45
t30	14.27 ± 0.98	12.92 ± 0.75
t60	16.51 ± 1.02	16.07 ± 1.05
t90	17.39 ± 1.30	16.30 ± 1.21
t120	15.27 ± 1.38	14.49 ± 1.17

**Table 4:** Mean values of blood glucose incremental area under the curve (AUCi), glucose maximum concentration (Cmax) and variation of glucose maximum concentration (∆Cmax) of study participants for intervention (*n* = 18) and control (*n* = 18) group.

Parameters	Intervention Group Mean ± SEM	Control Group Mean ± SEM	*p*-Value
AUCi_0–120min_ (mmol/L)	781.60 ± 53.81	798.45 ± 58.70	0.834 ^1^
Cmax (mmol/L)	18.19 ± 1.16	17.21 ± 0.99	0.527 ^1^
∆Cmax	10.09 ± 0.72	10.25 ± 0.67	0.873 ^1^

^1^*p*-value calculated by Mann–Whitney test between groups.

**Table 5:** Mean values of total polyphenols content and antioxidant capacity of aqueous cinnamon extract. The results are expressed in mean ± SEM.

Chemical Parameters	Aqueous Cinnamon Extract
Total Phenols (*n* = 3), mg/L Equivalent of Gallic acid ^1^	1554.9 ± 72.8
Antioxidant capacity—FRAP assay (*n* = 4), µmol Trolox/L ^2^	3658.8 ± 16.7
Antioxidant capacity—DPPH assay (*n* = 6), µmol Trolox/L ^3^	5125.0 ± 74.3

^1^ Linear equation: *y* = 0.0054*x* + 0.0267 (r² = 0.997); ^2^ Linear equation: *y* = 0.002*x* − 0.0022 (r² = 0.999); ^3^ Linear equation: *y* = −0.0012*x* + 1.0495 (r² = 0.999)

## Data Availability

The data presented in this study are available on request from the last author.
